# Parent Training Programs for Ethnic Minorities: a Meta-analysis of Adaptations and Effect

**DOI:** 10.1007/s11121-016-0733-5

**Published:** 2016-11-23

**Authors:** K. van Mourik, M. R. Crone, M. S. de Wolff, R. Reis

**Affiliations:** 1 Public Health and Primary Care, Leiden University Medical Center, P.O. Box 9600, 2300 RC Leiden, The Netherlands; 2 TNO Child Health, Leiden, The Netherlands; 3 Amsterdam Institute for Social Science Research, University of Amsterdam, Amsterdam, The Netherlands; 4 School of Child and Adolescent Health, the Children’s Institute, University of Cape Town, Cape Town, South Africa

**Keywords:** Meta-analysis, Cultural adaptation, Ethnic minority, Parenting behavior, Intervention

## Abstract

This meta-analysis focuses on parent training programs for ethnic minority families and reports on (i) the adaptation of program content and (ii) the process that informs these adaptations. Relevant studies are reviewed to determine the adaptations made and the impact of the adaptations on parenting and child outcomes. Studies were eligible for inclusion if they enrolled predominantly ethnic minority parents with children aged 0–12 years, used a randomized controlled trial design with post-intervention assessments, focused on group-based parent training programs and on prevention of parenting problems, and reported parenting behavior outcomes. A total of 18 studies were included in the analysis. The results show that parent training programs targeting ethnic minority parents have a small but significant effect on improving parenting behavior (*k* = 18, Cohen’s *d* = 0.30), child outcomes (*k* = 16, Cohen’s *d* = 0.13), and parental perspectives (*k* = 8, Cohen’s *d* = 0.19). Most of the programs made adaptations related to surface and deep structure sensitivity. Programs with cultural adaptations, especially deep structure sensitivity (*k* = 7, Cohen’s *d* = 0.54), are more effective in improving parenting behavior. Because only a third of the included studies provided details on the processes that guided the adaptations made, additional studies are needed to provide information on the process of adaptation; this will enable others to learn from the procedures that can be undertaken to culturally adapt interventions.

**Electronic supplementary material:**

The online version of this article (doi:10.1007/s11121-016-0733-5) contains supplementary material, which is available to authorized users.

Several studies have tested the efficacy of parent training programs to prevent parenting problems in varying subgroups, and, generally, such programs have a positive influence on parenting skills and child behavior (e.g.,: Bakermans-Kranenburg et al. 2003; Kaminski et al. 2008). However, the effectiveness of parent training programs varies based on participants’ characteristics and features of the parent training programs (Lundahl et al. 2006). Former studies indicate that an ethnic minority status is associated with poorer outcomes (Griner and Smith 2006; Lundahl et al. 2006). Researchers warn of the dangers of disseminating evidence-based interventions among diverse populations if such programs were originally developed according to European-American cultural norms and expectations (Cardona et al. 2012) and emphasize the relevance of cultural sensitivity and ecological validity for treatment and outcomes (Bernal et al. 2009).

With increased knowledge on evidence-based interventions, attempts have been made to adapt and develop parent training programs for ethnic minority groups (Baumann et al. 2015). Earlier meta-analysis indicates that by integrating factors such as language, cultural beliefs and explanatory models into the intervention, culture-sensitive programs may improve the target population’s acceptance and the effectiveness of the intervention (Griner and Smith 2006; Smith et al. 2011). However, there is a lack of evidence to guide practitioners on how best to culturally adapt interventions (Sidhu et al. 2015). The aim of the present meta-analysis was twofold. First, the included studies were reviewed to determine (a) the *type* of adaptations made to the parent training programs and (b) the extent to which the *process* that informed the adaptations is described. Second, we examined the influence of the adaptations on program effectiveness, i.e., the extent to which parenting problems were prevented in ethnic minority families.

## Cultural Adaptation Frameworks

There is no single correct way to culturally adapt parent training programs and no rule stating that every evidence-based intervention should be adapted (Baumann et al. 2015; Domenech Rodriguez et al. 2011). When considering cultural adaptations, frameworks providing guidance may inform the adaptation of either the program content, or the process of adaptation (Ferrer-Wreder et al. 2012). One such framework focusing on the *content* of the adaptation (in other words, on *what* to adapt) is the cultural sensitivity model (Resnicow et al. 1999). This distinguishes between surface and deep structure adaptations. Surface structure sensitivity involves matching program materials and messages to the characteristics of the target population (e.g., language, locations, and people). Deep structure sensitivity refers to incorporating elements that influence the behavior of the target group (e.g., cultural, social, and environmental factors). Frameworks directed at the process of adaptations guide decisions about *when* and *how* to adapt and *which* stakeholders to involve in the process. These models generally recommend to incorporate the expertise of stakeholders to inform the adaptations, the use of formative research, and to perform evaluation studies of the adapted intervention (Baumann et al. 2015; Domenech Rodriguez et al. 2011).

The present study combines a qualitative synthesis regarding the process and content of cultural adaptations, with a meta-analysis examining how adaptations impact the outcomes and effectiveness. We assess whether studies that provide information on the process of adaptation are more effective than those that do not describe the process of adaptation. This is based on the assumption that researchers that report the process of adaptations make better-informed choices resulting in improved outcomes in the target population. We also assess how the type of adaptations influences program effectiveness. It is reported that studies with more cultural adaptations are more effective than studies with fewer cultural adaptations (Smith et al. 2011). Based on the cultural sensitivity model of Resnicow et al. (1999), we assume that the impact is a result of the type of adaptations rather than the number of adaptations. Therefore, we compare programs with deep structure adaptations, surface structure adaptations, and no adaptations. Our hypothesis is that programs with deep structure adaptations will have enhanced effectiveness.

## Methods

### Study Selection

The parenting programs that are the focus of this meta-analysis are group-based interventions aimed at improving the quality of the parent–child relationship by changing parenting practices, aspects of parental functioning, and the child’s emotional and behavioral adjustment. The following seven criteria were applied for study inclusion: (1) the study sample consisted of predominantly (≥50%) ethnic minorities (i.e., a group of people who share a common culture, religion, language, or nationality (Hughes et al. 2006); we focus on ethnic groups that are minority groups in the country in which the intervention was delivered); (2) the intervention participants had a child aged 0–12 years (preschool to elementary school); (3) the intervention focused on the prevention of parenting problems (i.e., universal, selective, or indicated preventive interventions); (4) the intervention was group based; (5) the study reported a measure of parenting skills or behavior; (6) the study reported immediate post-intervention assessments; and (7) the study was a randomized controlled trial.

Having a post-intervention assessment was chosen as an inclusion criterion because, with a preventive sample, long-term effects are generally more difficult to demonstrate because different processes and outcomes may be involved (Sandler et al. 2011). Recently, the number of preventive parenting programs has increased exponentially and policymakers emphasize the need for systematic development and evaluation of these programs (Bakermans-Kranenburg et al. 2003). Therefore, the present study focused on preventive parent training programs. Programs were excluded when they were intended for children or parents who displayed clinically apparent or diagnosed with mental health problems or problem behavior (i.e., above a clinical cutoff point) and when parenting skills and behavior outcomes were only related to lifestyle behavior (e.g., physical activity, diet, and substance use) or literacy behavior. Individual programs (home-visiting programs and programs with an individual format) were also excluded; since these programs are generally tailored to the individual needs of parents, cultural adaptations differ between parents. This makes it difficult to categorize these adaptations and compare them with the inbuilt or systematically planned adaptations in group programs (i.e., brief structured programs with a specific focus on parenting).

A computer search was made in PsycInfo, PubMed, Medline, Embase, Web of Science, Cochrane/Central, and Cinhal/Academic Search Premier for all studies published up to August 2015. The search was performed using the following terms in varying combinations: parent program, minority group, ethnic, low income, disadvantaged community, and randomized controlled trial. Initially, this strategy identified 1327 studies. Preliminary study selection was based on scrutiny of the abstracts, which were reviewed by KM and MC, to determine whether they met the inclusion criteria. The preliminary screening resulted in 167 studies, each of which was assessed by reading the full article. Any disagreements were resolved by consensus, and a third reviewer (MW) was consulted in case of uncertainty about inclusion. When studies did not report immediate post-intervention data or the data were incomplete, the authors were contacted to provide additional data. Finally, 18 studies were included in the meta-analysis. The supplementary material (Figure i, available online) describes the selection of the studies; Table 1 lists and provides details on the included studies.

**Table 1:** Characteristics of included studies

Study	Intervention			Participants		Moderators		Effect sizes		
	Program name	Number of sessions	Country	Target group	Child’s age	Adaptations^a^	Methodological rigor^b^	Parenting behavior	Child behavior	Parental perspectives
Bjørknes and Manger 2013	Parent Management Training–Oregon Model (PMTO)	18	Norway	Muslim immigrant mothers from Somalia and Pakistan, who described their child’s behavior as a conduct problem	3–9	D/D/B	0/0/0	0.24	0.30	–
Brotman et al. 2011	ParentCorps	13	USA	Parents of children enrolled in prekindergarten in schools in large urban school districts	4	D/L/E	0/0/0	0.44	–	–
Coard et al. 2007	Black Parenting Strengths and Strategies (BPSS)	12	USA	Low-income caregivers who self-identified as African American	5–6	D/D/E	0/0/1	1.22	0.54	–
Day et al. 2012	Empowering Parents, Empowering Communities	8	UK	Socially disadvantaged families who identified difficulties in managing the child’s behavior	2–11	S/L/B	0/0/0	0.57	0.30	0.16
Fagan and Stevenson 2002	Men as Teachers	6	USA	African American fathers of children enrolled in services for low-income families	5.9 (mean)	D/L/B	1/0/1	0.43	–	0.31
Ghosh Ippen 1999	(No name)	9	USA	Low-income caregivers who self-identified as Latino	8–11	S/L/E	1/1/1	0.29	0.08	–
Gottfredson et al. 2006	Strengthening Washington DC Families Project	14	USA	Parent’s living in high-risk neighborhoods	0–12	B/L/E	1/1/0	0.00	−0.04	–
Gross et al. 2003	Incredible Years Parenting Program	12	USA	Parents of children enrolled in day care centers that serve low-income families	2–3	S/L/E	0/1/0	0.50	0.12	0.42
Gross et al. 2009	Chicago Parent Program	12	USA	Parents of children enrolled in day care centers that serve low-income families	2–4	S/D/E	0/0/0	−0.07	−0.02	−0.04
Kim et al. 2008	Incredible Years Parenting Program	12	USA	First generation Korean American mothers	3–8	S/L/B	0/0/1	0.10	0.01	–
Kim et al. 2014	Korean Parent Training Program (KPTP)	12	USA	First generation Korean American mothers	3–8	D/D/B	0/0/1	0.76	0.51	0.51
Lau et al. 2011	Incredible Years Parenting Program	14	USA	Chinese American parents with concerns about parenting or child behavior problems	5–12	D/L/E	0/0/1	0.84	0.76	0.40
Leijten et al. 2015	Incredible Years Parenting Program	12 to 18^c^	The Netherlands	Mothers experiencing parenting difficulties due to disruptive child behavior, recruited from outpatient clinics for child and adolescent psychiatry and in deprived neighborhoods	3–8	S/L/E	0/0/0	0.32	0.34	0.07
Matsumoto et al. 2007	Triple P Positive Parenting Program	8	Australia	Japanese Australian caregivers	2–10	S/L/B	0/0/1	0.25	0.38	0.47
Taylor et al. 1997	Group Well Child Care	7	USA	Mothers with one or more risk factors: poverty, single marital status at delivery, less than high school education, age less than 20 years at delivery, previous substance abuse or history of abuse as a child.	0	B/L/B	1/0/0	0.16	0.11	–
Turner et al. 2007	Triple P Positive Parenting Program	8	Australia	Australian indigenous families with concerns about the child’s behavior or parenting skills	1–13	D/L/B	0/0/1	0.56	0.15	–
Webster-Stratton 1998	PARTNERS	8 to 9	USA	Mothers of children enrolled in day care centers for low-income families	4	S/L/B	1/0/0	0.33	0.10	–
Webster-Stratton et al. 2001	Incredible Years Parenting Program	12	USA	Mothers of children enrolled in day care centers for low-income families	4	S/L/B	1/0/0	0.04	0.14	–
Overall effect size								0.30	0.13	0.18

^**a**^Moderators: cultural sensitivity (B = basic, S = surface structure, D = deep structure), process of adaptation (L = no to little information, D = detailed information), program delivery (B = basic, E = enhanced)

^**b**^Moderators: type of comparison condition (0 = no intervention, 1 = alternate intervention), intervention condition (0 = parent group alone, 1 = parent group plus additional intervention), sample size (0 = more than 35 per condition, 1 = 35 or less per condition)

^c^During the study, the number of sessions for the program was extended by the developer

### Outcome Measures

Preventive parent training programs generally operate under the premise that changes in parenting behavior will also affect child outcomes. Therefore, dependent measures in studies most often include some combination of child and parent outcomes. In the present study, as a primary outcome, changes in parenting behavior were examined. As a secondary outcome, we investigated changes in parental perceptions and in child (psychosocial) behavior.

#### Primary Outcome

Effect sizes were computed from measures of parenting behavior based on parental self-reported and observed measures. Most studies used only self-reported measures, whereas some combined both self-reported measures and observations of parent/child interactions. A diverse range of scales, subscales, and revised scales was reported by the studies (see supplementary material: Table ii, available online). Self-reported measures generally included broad band instruments, such as the Parenting Scale (PS: Arnold et al. 1993). Observational methods included (among others) the Dyadic Parent–Child Interactive Coding System-Revised (DPICS: Robinson and Eyberg 1978).

#### Secondary Outcomes

Measures of parental perceptions of parenting were derived from self-reported instruments, such as the Parenting Stress Index (PSI: Abidin 2015). The included child outcomes were related to child psychosocial development. Measures of child psychosocial behavior generally involved parent-reported measurements, whereas some studies combined self-reported measures and observations of parent/child interactions. The Eyberg Child Behavior Inventory (ECBI: Eyberg and Robinson 1983) was often reported as a measure of child psychosocial behavior. Examples of observational methods used by researchers included the Dyadic Parent–Child Interactive Coding System (DPICS: Robinson and Eyberg 1978).

### Effect Sizes

Cohen’s *d* was used as a measure of the effect size, which represents the difference between the treatment and control groups expressed in standard deviation units (Lipsey and Wilson 2001). The effect sizes and the 95% confidence intervals (CI) around the point estimates of the effect sizes were calculated using the software program CMA (Borenstein et al. 2000). Three meta-analytical data files were constructed, one for each outcome measure (i.e., parenting behavior, child behavior, and parental perspectives). Studies frequently reported results on multiple outcome measures and, incidentally, on multiple treatment conditions. To avoid overrepresentation of any study with multiple effect sizes or multiple treatment conditions, we aggregated the effect sizes within one study (Lipsey and Wilson 2001). In the case of more than one outcome measure for parenting behavior, child behavior, or parental perspective, the effect sizes were computed separately for each outcome and then aggregated. When studies included two or more intervention arms (e.g., a parent group, a child group, and a teacher group), effect sizes were calculated for the effects of the treatment conditions with the parent group component compared to the control condition.

### Moderators

Moderator analyses were used to provide a more specific assessment of the effects based on the study characteristics expected to influence outcomes. Two characteristics of adaptations made to parent training programs were coded to assess their potential role as moderating variables: (1) cultural sensitivity and (2) process of adaptation. Cultural sensitivity was coded as “basic,” “surface structure sensitivity,” or “deep structure sensitivity.” Parent training programs were coded “basic” if studies did not report adaptations related to the participant group. The code “surface structure sensitivity” was given when programs adapted program materials and messages to characteristics of the target population (e.g., language, people, location). The code “deep structure sensitivity” was given when programs incorporated elements that influence the parenting behavior of the target group (e.g., content related to cultural, social, and environmental factors). Programs were coded to include detailed information on the process of adaptation and whether the study described the way in which stakeholders’ expertise, data from qualitative or quantitative research, or formal evaluation of the program, informed the adaptations made. See the supplementary material (Table ii, available online) for more information on the coding of the above mentioned moderators. Three characteristics of methodological rigor were coded as indicators of internal validity: (i) type of comparison group, (ii) intervention condition, and (iii) sample size. Based on the study of Lipsey and Wilson (2001), who analyzed a large amount of meta-analyses and provided information on study indicators related to methodological rigor, two moderators were created. The type of comparison group in each study was coded as “control group received no intervention” (including waitlist control group) or “control group received an alternate intervention.” The precise intervention condition was coded as “parent group alone” if the parent training program was evaluated as a stand-alone program, or as “parent group plus additional interventions” if the parent training program was evaluated as a part of a wider array of interventions (Table 2). The third moderator related to methodological rigor was the number of participants in the intervention and control condition. Fewer than 35 participants in a condition was considered a small sample size because studies with small sample sizes are known to be positively biased, thereby risking overestimation of the magnitude of effect sizes (Hertzog 2008).

**Table 2:** Coding system for moderators

Variable	Coding description
Adaptations	
Cultural sensitivity	B = basic parent training program
	S = surface structure sensitivity (i.e., matching materials and messages)
	D = deep structure sensitivity (i.e., content adaptations)
Process of adaptation	L = no to little information
	D = detailed information (i.e., description how process of adaptations was informed)
Methodological rigor	
Type of comparison group	0 = control group received no intervention
	1 = control group received an alternate intervention
Intervention condition	0 = parent group alone
	1 = parent group plus additional intervention
Sample size	0 = more than 35 participants per intervention condition
	1 = 35 or less participants per intervention condition

The moderators were tested for homogeneity using the within-class goodness-of-fit statistic (Johnson 1993). A significant Q_within_ statistic suggests heterogeneity within a set of studies and the need for a moderator analysis. The influence of moderators was calculated using the Q statistic for between-group differences. The Q_between_ statistic indicates significant differences between the subgroups of a moderator.

### Reliability

The first author (KM) coded all studies. A trained graduate student coded a random sample of nine of the 18 studies. Cohen’s kappa values were computed for the two categories of moderators, i.e., adaptations and methodological rigor. This resulted in kappa values of 0.55 and 1.00, respectively. These kappa values can be interpreted as a moderate to very good agreement (Viera and Garrett 2005). Disagreements were resolved by discussions among the coders and, when necessary, were discussed with a third researcher (MC) until consensus was reached.

### Publication Bias

To address possible publication bias, funnel plots and the fail-safe N were examined (Rosenthal 1979). A funnel plot is a plot of each study based on its effect size and standard error. The plot is expected to show a funnel shape with a symmetrical distribution on the right and left sides of the mean if there is no publication bias. The fail-safe N represents the number of studies required to nullify the intervention effect, i.e., the number of additional studies in which the intervention effect was zero that is needed to increase the *p* value for the meta-analysis above 0.05.

## Results

### Study Descriptives

Table 1 provides an overview of the characteristics of the parent training programs. Overall, there was a high percentage of ethnic minorities (range 60–100%) in the study samples. This was the result of the intentional recruitment of parents from ethnic groups (Bjørknes and Manger 2013; Coard et al. 2007; Fagan and Stevenson 2002; Ghosh Ippen 1999; Kim et al. 2008, 2014; Lau et al. 2011; Matsumoto et al. 2007; Turner et al. 2007) or (for studies targeting parents from deprived neighborhoods) the multi-ethnic composition of these neighborhoods (Brotman et al. 2011; Day et al. 2012; Gottfredson et al. 2006; Gross et al. 2003; Gross et al. 2009; Leijten et al. 2015; Taylor et al. 1997; Webster-Stratton 1998; Webster-Stratton et al. 2001).

### Cultural Sensitivity

Of the 18 studies, 16 made adaptations of an existing intervention or created a new culturally tailored program specifically for ethnic minority families (Bjørknes and Manger 2013; Brotman et al. 2011; Coard et al. 2007; Day et al. 2012; Fagan and Stevenson 2002; Ghosh Ippen 1999; Gross et al. 2003; Gross et al. 2009; Kim et al. 2008, 2014; Lau et al. 2011; Leijten et al. 2015; Matsumoto et al. 2007; Turner et al. 2007; Webster-Stratton 1998; Webster-Stratton et al. 2001). Below, we first describe what adaptations were made: categorized as surface structure sensitivity and deep structure sensitivity. Second, we describe what information was provided regarding the process of adaptation.

#### Surface Structure Adaptations

Adaptations in *language* were made in eight studies (Bjørknes and Manger 2013; Ghosh Ippen 1999; Kim et al. 2014; Kim et al. 2008; Lau et al. 2011; Leijten et al. 2015; Matsumoto et al. 2007; Webster-Stratton et al. 2001). These adaptations mainly consisted of the translation of program materials. Interpreters or bilingual group leaders were used, and adaptations were made with regard to proverbs and language expression in a minority of studies (Bjørknes and Manger 2013; Coard et al. 2007; Leijten et al. 2015).

Adaptations in *persons* were made in 12 studies. Matching of group leaders with the characteristics of the participants was reported in seven studies, based on ethnicity (Coard et al. 2007; Gross et al. 2003; Gross et al. 2009; Turner et al. 2007) or with the use of local parents as peer facilitators (Day et al. 2012; Fagan and Stevenson 2002; Webster-Stratton 1998). The use of bicultural group leaders who could function as cultural brokers was explicitly mentioned in three studies (Bjørknes and Manger 2013; Kim et al. 2014; Matsumoto et al. 2007). Three studies paid attention to the homogenization of gender. In two studies, the program was provided to mother-only groups to meet the cultural norms of Muslim women (Bjørknes and Manger 2013; Leijten et al. 2015), and the third used only male staff in a program provided to fathers (Fagan and Stevenson 2002).

Changes in program *materials* were mentioned in the seven studies (Bjørknes, R and Manger 2013; Brotman et al. 2011; Gross et al. 2009; Leijten et al. 2015; Turner et al. 2007; Webster-Stratton 1998; Webster-Stratton et al. 2001). Adaptations were made to manuals and video segments to portray familiar and relevant situations for parents, e.g., images in workbooks and video segments depicting families from a variety of ethnic backgrounds (Brotman et al. 2011; Gross et al. 2009; Turner et al. 2007; Webster-Stratton 1998; Webster-Stratton et al. 2001). To overcome language barriers, pictures were added to the homework assignments (Leijten et al. 2015) and role-playing was added to the sessions (Bjørknes and Manger 2013).

#### Deep Structure Adaptations

Deep structure adaptations were made in seven studies, in addition to surface structure adaptations (Bjørknes and Manger 2013; Brotman et al. 2011; Coard et al. 2007; Fagan and Stevenson 2002; Kim et al. 2014; Lau et al. 2011; Turner et al. 2007). Deep structure sensitivity included sessions with specific content, which paid attention to large sibling groups, emotion control, racial socialization, cultural and contextual influences on parenting, and communication training (Bjørknes and Manger 2013; Brotman et al. 2011; Coard et al. 2007; Fagan and Stevenson 2002; Lau et al. 2011). More time was allowed to discuss cultural and contextual (e.g., social, political) influences on parenting in two studies (Brotman et al. 2011; Turner et al. 2007). One study reported making the overall program culturally sensitive, by incorporating cultural and religious elements and parenting norms (Kim et al. 2014).

### Process of Adaptation

Detailed information on the process that guided the adaptations was given in five studies, of which three had surface and deep structure sensitivity (Bjørknes and Manger 2013; Coard et al. 2007; Kim et al. 2014), and one study had surface structure sensitivity (Gross et al. 2009). Two studies referred to recent literature and described how this influenced the program development (Coard et al. 2007; Kim et al. 2014), one study used information from a former pilot study to adapt the parent training program (Bjørknes and Manger 2013), and another study provided details on the involvement of an advisory board and how this influenced the program development (Gross et al. 2009).

Twelve studies with surface or deep structure sensitivity did not offer detailed information on the process of adaptation. Three studies reported collaboration with local stakeholders and information from a pilot study, without providing details on how this guided the process of adaptation of the program (Brotman et al. 2011; Fagan and Stevenson 2002; Turner et al. 2007). Other studies provided no information on the process of adaptation (Day et al. 2012; Ghosh Ippen 1999; Gross et al. 2003; Kim et al. 2008; Lau et al. 2011; Leijten et al. 2015; Matsumoto et al. 2007; Webster-Stratton 1998; Webster-Stratton et al. 2001).

### Effect Sizes and Moderator Analysis

Effect sizes regarding the parenting behavior construct, child behavior construct, and parental perspective construct were calculated for 18 studies, 16 studies, and 8 studies, respectively. Table 1 provides an overview of effect sizes per study and the overall effect per outcome construct.

### Primary and Secondary Outcomes

Effect sizes for parenting behavior ranged from −0.07 to 1.22, with an overall effect size of 0.30 (95% CI 0.17–0.44). This reflects a small but significant difference between the intervention and comparison groups at immediate post-intervention. The parenting behavior construct contained significant heterogeneity (Q_within_ = 34.79, *p* < 0.05), which implies the relevance for moderator analysis. Effect sizes for child behavior ranged from −0.04 to 0.76, with an overall effect size of 0.13 (95% CI 0.05–0.22). Effect sizes for parental perspectives ranged from −0.04 to 0.47, with an overall effect size of 0.19 (95% CI 0.04–0.35). This reflected a small difference between the intervention groups, favoring the intervention group. Homogeneity was tested and was found to be non-significant for child behavior (Q_within_ = 15.72, *p* > 0.05) and parental perspectives (Q_within_ = 7.80, *p* > 0.05), indicating no marked variability in reported effect sizes across studies. Therefore, Table 3 presents the results of the moderator analysis for the primary outcome, whereas results of the moderator analysis for the secondary outcomes are described only in the text.

**Table 3:** Moderators associated with parenting behavior outcomes

	Parenting behavior			
Adaptations	*d*	*k*	Q_within_	Q_between_
Cultural sensitivity				8.05*
Basic	0.10	3	2.59	
Surface structure	0.24	10	13.90	
Deep structure	0.54	7	6.59	
Process of adaptation				0.07
No to little information	0.29	15	21.13	
Detailed information	0.35	5	13.19**	
Methodological rigor	d	k	Q_within_	Q_between_
True “no” treatment control group				2.56
Yes	0.39	13	23.03*	
No	0.17	7	7.91	
Parent training program evaluated as stand-alone program				0.38
Yes	0.31	16	26.10*	
No	0.23	4	6.95*	
Sample size smaller than 35 per condition				5.04*
Yes	0.52	8	7.44	
No	0.22	12	20.00*	

*d* effect size, *k* number of studies, *C.I.* confidence interval, *Q*_*within*_ within-class goodness-of fit

Q_between_ = between-group goodness-of fit

*p < 0.05, **p < 0.01

### Moderator Analysis

Cultural sensitivity was significantly related to effect size (Q_between_ = 8.61, *p* < 0.05). The highest effect size was found for studies with deep structure sensitivity (*d* = 0.54), indicating that the parenting behavior of parents in programs with deep structure sensitivity improved the most. An exploratory moderator analysis indicated that a significant effect was also found for the child behavior construct (Q_between_ = 8.13, *p* < 0.05). Contrary to our expectation, process of adaptation had no significant influence on parenting outcomes.

The second set of moderators examined indicators of methodological rigor to ascertain the extent to which effect sizes reflect the impact of the parent training program, rather than the methodological influences or biases. One of the three rigor moderators predicted significant differences in effect sizes for parenting behavior. Studies with a sample size of ≤35 per condition had a significantly higher effect for the parenting behavior construct (Q_between_ = 4.62, *p* < 0.05).

### Publication Bias

A funnel plot was examined for the parenting behavior construct, which was asymmetrical, with a gap in the bottom left-hand corner. This indicates that the true effect might be smaller than that found, due to unpublished studies with non-significant findings. A calculation of the fail-safe N revealed that 180 unpublished studies with non-significant results would have to exist to reduce the level of significance of the effect size for parenting behavior to a *p* value ≥ 0.05.

## Discussion

This study includes a wide variety of preventive parent training programs delivered to ethnic minority families. The aim was to (i) describe the type of cultural adaptations made and (ii) the process that informed the adaptations. In addition, the influence of the adaptations on program effectiveness was examined.

The current meta-analysis shows the effectiveness of parent training programs for parenting behavior and, to a lesser extent, for parental perspectives and child behavior. The majority of studies made adaptations to the program, consisting of surface structure adaptations (related to language, persons, and program materials) and deep structure adaptations (related to specific content and cultural/contextual influences and norms). As expected, there was a significant positive effect supporting the relevance of making parent training programs culturally sensitive. Programs with cultural sensitivity were more successful in improving parenting behavior compared with interventions without cultural sensitivity. These findings are in line with earlier studies showing that culturally adapted treatments were more effective than non-adapted treatments (Griner and Smith 2006; Smith et al. 2011). Our study reveals that cultural sensitivity can be achieved in different ways, and it was found that, in particular, deep structure sensitivity resulted in a program effect. These findings may provide a different perspective on former studies indicating that the most effective treatments were those with more adaptations (Griner and Smith 2006; Smith et al. 2011). Analyses were done on a study level, which implies that cultural sensitivity may not be an integral element of the original parent training program, but rather a one-time adjustment or aspect of program implementation. Also, the topic is subject to report bias, implying that adaptations were only coded when specifically mentioned in the included studies.

Only a third of the studies with cultural sensitivity provided information on the process that guided the adaptations made. This is in line with Baumann et al. (2015), who found that few studies provided information on how choices were made regarding adaptations and which stakeholders were involved. Although the process of adaptation was not found to influence program effectiveness, we agree with others in the field of cultural adaptation that it is important to document this process. Detailed information on the process of adaptations enables researchers and professionals to identify the range of adaptations that can be made and provides insight into the relevant procedures that could be undertaken in any process of cultural adaptation (Cardona et al. 2012; Ferrer-Wreder et al. 2012; Griner and Smith 2006). A requirement for publication should be that information is provided about the process that guided the adaptations. Transparency is important because adaptations are sometimes based on perceptions of what ethnic minority parents want and a need to be politically correct, as opposed to based on empirical evidence (Morawska et al. 2011).

### Strengths and Limitations

A limitation of the present study is that a multivariable approach was not possible due to higher number of studies required to perform this approach. Our moderator analysis indicated that programs with cultural sensitivity were more effective; however, the presence of (confounding) study or sample characteristics might account for the differences found. Therefore, further research is required on this topic. Another limitation is the heterogeneity of outcomes in the parent training programs. The studies used a variety of validated and not validated instruments, as well as different outcome constructs for parenting and child behavior. We advocate the use of more standardized and validated instruments and specifically discourage the construction of outcomes by using elements of existing instruments and scales. This not only makes study outcomes less transparent, but it also hinders the comparability between studies.

The present study analyzed the impact of methodological rigor. Sample size was found to be a moderator for effect, with stronger effect sizes in studies with small sample sizes. This might be the result of publication bias, because studies with non-significant results are less likely to be published. Based on the fill and trim method, potential publication bias does not appear to be a substantial threat to the results obtained in our meta-analysis. Griner and Smith (2006) mention inadequate funding for the larger-scale studies that are required to provide solid empirical evidence, as a hindrance to further development in the field of cultural adaptation.

### Implications

Although the present study focuses on programs that target predominantly ethnic minority populations, the study samples often consisted of populations with both an ethnic minority status and a low socioeconomic status (SES). When implementing a parent training program in multi-ethnic deprived neighborhoods, one needs to be sensitive to the diversity of the target group. Ethnic minority and a low SES often coincide (Costello et al. 2001), which argues for adaptations that are sensitive across cultures. An alternative to adaptations (i.e., inbuilt changes to an intervention) is to focus on sensitivity in which interventions have an explicit inbuilt flexibility to allow professionals and parents to adjust the program elements to their specific values and norms (Mejia et al. 2016).

Our findings show that the preventive effect of parent training programs on outcomes is small but positive. It is important to note that higher initial levels of behavioral problems generally predict greater improvement, due to a larger scope for improvement and more motivation to change (Leijten et al. 2013). Caulkins et al. (2004) showed that, in preventive interventions, even a small effect size can lead to important savings for society when large groups are reached. It is reported that ethnic minorities and disadvantaged families are more difficult to recruit and retain (Furlong et al. 2012). Therefore, it is a challenging task for policymakers and providers of support to recruit ethnic minority families in the most optimal way. Although the present study does not provide information on how cultural adaptations are related to attendance rates, this is an interesting topic for future reviews.

## Conclusions

The majority of programs included in this meta-analysis made adaptations. Detailed information was provided on the type of adaptations and how this was related to program effect. The findings confirm the effectiveness of cultural sensitivity in parent training programs, with deep structure sensitivity resulting in the highest impact on parenting behavior. Only a few studies described the process that guided the adaptations made, which is a barrier to learning from relevant procedures that could be undertaken in the process of cultural adaptation. Program delivery (i.e., overcoming practical barriers for participation) did not appear to influence program effect.

## Electronic supplementary material

Below is the link to the electronic supplementary material.

ESM 1 (DOCX 134 kb)
