# Supplementary material for: Parent Training Programs for Ethnic Minorities: a Meta-analysis of Adaptations and Effect
Source: Prev Sci. 2016 Nov 23;18(1):95–105. doi: 10.1007/s11121-016-0733-5 (PMC5236066; doi:10.1007/s11121-016-0733-5)
Supplement: Supplementary file 1 — (DOCX 134 kb) [file 11121_2016_733_MOESM1_ESM.docx]

**Supplementary material**

Figure i Flow chart of inclusion

1327 records identified through database searching

1160 records excluded based on abstract

149 full text articles excluded

No group programme (n=47)

No parenting behavior outcome (n=32)

No preventive programme (n=17)

Not the target group (n=14)

No post-intervention assessment (n=10)

Incomplete data (n=7)

Other (n=22)

167 full-text articles assessed for eligibility

18 articles included

Table i Assessment instruments of included studies

| **Bjørknes et al. (2013)** | | |  | |
| --- | --- | --- | --- | --- |
| **Outcome name** | **Assessment instrument** | **Information about instrument ^[[1]](#footnote-1)^** | | **Reported by** |
| Parenting behavior | Discipline style: harsh (harsh discipline scale, Positive Practices Interview, PPI) | (Webster-Stratton, 1998a) | | Parent |
|  | Discipline style: harsh for age (harsh for age scale, Positive Practices Interview, PPI) | (Webster-Stratton, 1998a) | | Parent |
|  | Discipline style: appropriate discipline (appropriate discipline scale, Positive Practices Interview, PPI) | (Webster-Stratton, 1998a) | | Parent |
|  | Positive parenting (positive parenting scale, Positive Practices Interview, PPI) | (Webster-Stratton, 1998a) | | Parent |
| Child behavior | Problem behavior: conduct problems | Constructed variable, based on Eyberg Child Behavior Inventory intensity scale (ECBI) (Eyberg & Pincus, 1999) and Parent Daily Report (PDR) (Chamberlain & Reid, 1987) | | Parent |
|  | Problem behavior (problem scale, Eyberg Child Behavior Inventory, ECBI) | (Eyberg & Pincus, 1999) | | Parent |
| Parental perspectives |  |  | |  |

| **Brotman et al. (2011)** | | |  | |
| --- | --- | --- | --- | --- |
| **Outcome name** | **Assessment instrument** | **Information about instrument** | | **Reported by** |
| Parenting behavior | Discipline style (appropriate discipline and clear expectations scale, Parenting Practices Interview, PPI) | (Webster-Stratton, 1998a) | | Parent |
|  | Parenting practices: Effective behavior management practices (Effective Parenting Test, EPT) | (Calzada & Brotman, 2002) | | Parent |
|  | Parent-child interactions: Parenting effectiveness and scaffolding of child behavior (Global Impressions of Parent Child Interactions-Revised, GIPCI-R) | (Brotman et al., 2005; Brotman et al., 2007) | | Observer |
| Child behavior |  |  | |  |
| Parental perspectives |  |  | |  |

| **Coard et al. (2007)** | | |  | |
| --- | --- | --- | --- | --- |
| **Outcome name** | **Assessment instrument** | **Information about instrument** | | **Reported by** |
| Parenting behavior | Parenting practices: monitoring (monitoring scale, Parenting Practices Interview, PPI) | (Webster-Stratton, 1998a) | | Parent |
|  | Positive parenting (positive parenting scale, Parenting Practices Interview, PPI) | (Webster-Stratton, 1998a) | | Parent |
|  | Discipline style: harsh (harsh discipline scale, Parenting Practices Interview, PPI) | (Webster-Stratton, 1998a) | | Parent |
|  | Racial socialization (Parenting Experience of Racial Socialization, PERS) | (Stevenson, 1994) | | Parent |
| Child behavior | Problem behavior: conduct problems (conduct problems scale, Behavioral Assessment System of Children-Parent, BASC-P) | (Reynolds & Kamphaus, 1992, 2002) | | Parent |
|  | Social skills: responsibility (responsibility scale, Social Skills Rating Scale-Parent, SSRS-P) | (Gresham & Elliot, 1990) | | Parent |
|  | Social skills: cooperation (cooperation scale, Social Skills Rating Scale-Parent, SSRS-P) | (Gresham & Elliot, 1990) | | Parent |
|  | Social skills: self-control (self-control scale, Social Skills Rating Scale-Parent, SSRS-P) | (Gresham & Elliot, 1990) | | Parent |
| Parental perspectives |  |  | |  |

| **Day et al. (2012)** | | |  | |
| --- | --- | --- | --- | --- |
| **Outcome name** | **Assessment instrument** | **Information about instrument** | | **Reported by** |
| **Parenting behavior** | Parenting practices (laxness scale, overreactivity scale and verbosity scale, Arnold O'Leary parenting scale) | (Arnold, O'Leary, Wolff, & Acker, 1993) | | Parent |
| **Child behavior** | Problem behavior (problem scale, Eyberg Child Behavior Inventory, ECBI) | (Eyberg & Ross, 1978) | | Parent |
|  | Problem behavior: intensity (intensity scale, Eyberg Child Behavior Inventory, ECBI) | (Eyberg & Ross, 1978) | | Parent |
|  | Problem behavior (total score, Strengths and Difficulties Questionnaire, SDQ) | (Goodman, 1997) | | Parent |
|  | Problem behavior: conduct problems (conduct score, Strengths and Difficulties Questionnaire, SDQ) | (Goodman, 1997) | | Parent |
|  | SDQ: hyperactivity/inattention (hyperactivity/inattention score, Strengths and Difficulties Questionnaire, SDQ) | (Goodman, 1997) | | Parent |
|  | Concerns about the child (Concerns about my child measure) | (Scott, Spender, Doolan, Jacobs, & Aspland, 2001) | | Parent |
| **Parental perspectives** | Parenting stress (Parenting Stress Index – short form) | (Abidin, 1995) | | Parent |

| **Fagan et al. (2002)** | | |  | |
| --- | --- | --- | --- | --- |
| **Outcome name** | **Assessment instrument** | **Information about instrument** | | **Reported by** |
| Parenting behavior | Racial socialization (racial oppression scale, Racial socialization - Parent version) | (Stevenson, 1997) | | Parent |
| Child behavior |  |  | |  |
| Parental perspectives | Satisfaction with parental role (satisfaction scale, Self-perceptions of the Parental Role) | (MacPhee, Benson, & Bullock, 1986) | | Parent |

| **Gottfredson et al. (2006)** | | |  | |
| --- | --- | --- | --- | --- |
| **Outcome name** | **Assessment instrument** | **Information about instrument** | | **Reported by** |
| Parenting behavior | Parenting practices | Constructed variable: based on family cohesion scale, organization scale and conflict scale, Moos Family Environment Scale, FES (Moos & Moos, 1986), confidence in parenting based on measures used in Kumpfer's evaluations of SFP (no reference was made to an article) and consistency in discipline scale (modelled after Oregon youth Study, cited in Gottfredson, Harmon, Gottfredson, Jones, & Celestin, 1996) | | Parent |
| Child behavior | Problem behavior | Constructed variable, based on hyperactivity/impulsivity scale and antisocial behavior scale of the Parent Observation of Children's Activities, POCA-R (Kellam, 1990) and Social Skills Rating System, SSRS (Gresham & Elliot, 1990) | | Parent |
|  | Child positive adjustment | Constructed variable, based on social skills scale, school progress scale and sociability scale of the Parent Observation of Children's Activities, POCA-R (Kellam, 1990) and Social Skills Rating System, SSRS (Gresham & Elliot, 1990) | | Parent |
| Parental perspectives |  |  | |  |

| **Gross et al (2003)** | | |  | | | |
| --- | --- | --- | --- | --- | --- | --- |
| **Outcome name** | **Assessment instrument** | **Information about instrument** | | | | **Reported by** |
| Parenting behavior | Discipline style (coercive discipline strategies scale, Parenting Scale) | (Arnold et al., 1993) | | | | Parent |
|  | Parent-child interactions: total commands (Dyadic Parent-Child Interactive Coding System-Revised, DPICS-R) | (Webster-Stratton, 1985b, 1998b) | | | | Observer |
|  | Parent-child interactions: positive parenting (Dyadic Parent-Child Interactive Coding System-Revised, DPICS-R) | (Webster-Stratton, 1985b, 1998b) | | | | Observer |
| Child behavior | Problem behavior (problem scale, Eyberg Child Behavior Inventory, ECBI) | (Robinson, Eyberg, & Ross, 1980) | | | | Parent |
|  | Problem behavior: intensity (intensity scale, Eyberg Child Behavior Inventory, ECBI) | (Robinson et al., 1980) | | | | Parent |
|  | Problem behavior: oppositional (oppositional factor of the intensity scale, Eyberg Child Behavior Inventory, ECBI) | (Robinson et al., 1980) | | | | Parent |
|  | Problem behavior: inattentive (inattentive factor of the intensity scale, Eyberg Child Behavior Inventory, ECBI) | (Robinson et al., 1980) | | | | Parent |
|  | Problem behavior: conduct (conduct factor of the intensity scale, Eyberg Child Behavior Inventory, ECBI) | (Robinson et al., 1980) | | | | Parent |
|  | Parent-child interactions: problem behavior (Dyadic Parent-Child Interactive Coding System-Revised, DPICS-R) | (Webster-Stratton, 1998b) | | | | Observer |
| Parental perspectives | Parenting self-efficacy (parent's self-efficacy scale, Toddler Care Questionnaire, TCQ) | (Gross, Conrad, Fogg, Willis, & Garvey, 1995; Gross & Rocissano, 1988) | | | | Parent |
|  |  |  |  |  |  |  |

| **Gross et al. (2009)** | | |  | |
| --- | --- | --- | --- | --- |
| **Outcome name** | **Assessment instrument** | **Information about instrument** | | **Reported by** |
| Parenting behavior | Discipline style: following through on discipline (subscale following through on discipline, adapted version of the Parenting Questionnaire, PQ) | Adapted version of the Parenting Questionnaire, PQ (McCabe, Clark, & Barnett, 1999) | | Parent |
|  | Parenting practices: warmth (subscale warmth, adapted version of the Parenting Questionnaire, PQ) | Adapted version of the Parenting Questionnaire, PQ (McCabe et al., 1999) | | Parent |
|  | Discipline style: corporal punishment (subscale corporal punishment, adapted version of the Parenting Questionnaire, PQ) | Adapted version of the Parenting Questionnaire, PQ (McCabe et al., 1999) | | Parent |
|  | Parent-child interactions: positive parenting (during play session, Dyadic Parent-Child Interactive Coding System, DPICS-R) | (Webster-Stratton, 1998b) | | Observer |
|  | Parent-child interactions: positive parenting (during clean-up session, Dyadic Parent-Child Interactive Coding System, DPICS-R) | (Webster-Stratton, 1998b) | | Observer |
|  | Parent-child interactions: total commands (during clean-up session, Dyadic Parent-Child Interactive Coding System, DPICS-R) | (Webster-Stratton, 1998b) | | Observer |
|  | Parent-child interactions: total commands (during clean-up session, Dyadic Parent-Child Interactive Coding System, DPICS-R) | (Webster-Stratton, 1998b) | | Observer |
| Child behavior | Problem behavior (problem scale, Eyberg Child Behavior Inventory, ECBI) | (Eyberg & Pincus, 1999) | | Parent |
|  | Problem behavior: intensity (intensity scale, Eyberg Child Behavior Inventory, ECBI) | (Eyberg & Pincus, 1999) | | Parent |
|  | Parent-child interactions: aversive behavior (during play session, Dyadic Parent-Child Interactive Coding System, DPICS-R) | (Webster-Stratton, 1998b) | | Observer |
|  | Parent-child interactions: aversive behavior (during clean-up session, Dyadic Parent-Child Interactive Coding System, DPICS-R) | (Webster-Stratton, 1998b) | | Observer |
| Parental perspectives | Parenting self-efficacy (Toddler Care Questionnaire, TCQ) | (Gross & Rocissano, 1988) | | Parent |

| **Ghosh Ippen (1998)** | | |  | |
| --- | --- | --- | --- | --- |
| **Outcome name** | **Assessment instrument** | **Information about instrument** | | **Reported by** |
| Parenting behavior | Discipline style: nonaggressive punishment (Discipline Styles Questionnaire, DSQ) | Scale developed for study | | Parent |
|  | Discipline style: coercive punishment (Discipline Styles Questionnaire, DSQ) | Scale developed for study | | Parent |
| Child behavior | Problem behavior (problem scale, Eyberg Child Behavior Inventory, ECBI) | (Eyberg & Ross, 1978) | | Parent |
|  | Problem behavior: intensity (intensity scale, Eyberg Child Behavior Inventory, ECBI) | (Eyberg & Ross, 1978) | | Parent |
|  | Number of conflicts (Issues Checklist, IC) | (Prinz, Foster, Kent, & O'Leary, 1979) | | Parent |
|  | Frequency of conflict (Issues Checklist, IC) | (Prinz et al., 1979) | | Parent |
| Parental perspectives |  |  | |  |

| **Kim et al. (2008)** |  |  |  |
| --- | --- | --- | --- |
| **Outcome name** | **Assessment instrument** | **Information about instrument** | **Reported by** |
| Parenting behavior | Discipline style: positive discipline (positive discipline scale, Parenting Practices Interview, PPI) | (Webster-Stratton, 2007a) | Person |
|  | Discipline style: appropriate (appropriate discipline scale, Parenting Practices Interview, PPI) | (Webster-Stratton, 2007a) | Person |
|  | Discipline style: harsh (harsh discipline scale, Parenting Practices Interview, PPI) | (Webster-Stratton, 2007a) | Person |
| Child behavior | Problem behavior (problem scale, Eyberg Child Behavior Inventory, ECBI) | (Robinson et al., 1980) | Person |
|  | Problem behavior (intensity scale, Eyberg Child Behavior Inventory, ECBI) | (Robinson et al., 1980) | Person |
|  | Social skills: social competence (Social Competence Scale) | (Webster-Stratton, 2007b) | Person |
| Parental perspectives |  |  |  |

| **Kim et al. (2014)** | | | |
| --- | --- | --- | --- |
| **Outcome name** | **Assessment instrument** | **Information about instrument** | **Reported by** |
| Parenting behavior | Discipline style: harsh (harsh discipline scale, Korean Parent Discipline Interview, KPDI) | (Kim, Guo, Koh, & Cain, 2010) | Parent |
|  | Discipline style: positive discipline (positive discipline scale, Korean Parent Discipline Interview, KPDI) | (Kim et al., 2010) | Parent |
|  | Discipline style: Appropriate (appropriate discipline scale, Korean Parent Discipline Interview, KPDI) | (Kim et al., 2010) | Parent |
|  | Parenting practices: emotion coaching (Emotion Coaching Parenting Style) | (Gottman, Declaire, & Goleman, 1998) | Parent |
|  | Parenting practices: parental rejection (rejection scale, Parental Acceptance-Rejection Questionnaire, PARQ) | (Rohner, 1991) | Parent |
|  | Parenting practices: parental warmth (warmth scale, Parental Acceptance-Rejection Questionnaire, PARQ) | (Rohner, 1991) | Parent |
|  | Parent-child interactions: positive (Dyadic Parent-Child Interaction Coding System, DPIC) | (Eyberg & Robinson, 2000) | Observer |
|  | Parent-child interactions: negative (Dyadic Parent-Child Interaction Coding System, DPIC) | (Eyberg & Robinson, 2000) | Observer |
|  | Parent-child interactions: praises (Dyadic Parent-Child Interaction Coding System, DPIC) |  |  |
|  | Parenting practices: emotion coaching (Emotion Coaching Parenting Style) | (Gottman et al., 1998) |  |
| Child behavior | Problem behavior (Pediatric Symptoms Checklist) | (Jellinek & Murphy, 1990) | Parent |
| Parental perspectives | Parenting self-efficacy (Parenting Self-Efficacy Scale) | (Choe & Chung, 2010)Choe & Chung 2010 | Parent |

| **Lau et al. (2011)** | | |  | |
| --- | --- | --- | --- | --- |
| **Outcome name** | **Assessment instrument** | **Information about instrument** | | **Reported by** |
| Parenting behavior | Parenting practices: positive involvement (positive involvement scale, Alabama Parenting Questionnaire, APQ) | (Shelton, Frick, & Wootten, 1996) | | Parent |
|  | Discipline style: negative discipline (negative discipline scale, Alabama Parenting Questionnaire, APQ) | (Shelton et al., 1996) | | Parent |
| Child behavior | Problem behavior: internalizing problems (internalizing scale, Child Behavior CheckList, CBCL) | (Achenbach & Rescorla, 2001) | | Parent |
|  | Problem behavior: internalizing problems (externalizing scale, Child Behavior CheckList, CBCL) | (Achenbach & Rescorla, 2001) | | Parent |
| Parental perspectives | Parenting stress (Parenting Stress Index - short form, PSI-SF) | (Abidin, 1995) | | Parent |

| **Leijten et al. (2015)** | | |  | |
| --- | --- | --- | --- | --- |
| **Outcome name** | **Assessment instrument** | **Information about instrument** | | **Reported by** |
| Parenting behavior | Discipline style: appropriate discipline (Parenting Practices Interview, PPI) | (Webster-Stratton, 1998b; Webster-Stratton, Reid, & Hammond, 2004) | | Parent |
|  | Discipline style: harsh and inconsistent (Parenting Practices Interview, PPI) | (Webster-Stratton, 1998b; Webster-Stratton et al., 2004) | | Parent |
|  | Discipline style: physical punishment (Parenting Practices Interview, PPI) | (Webster-Stratton, 1998b; Webster-Stratton et al., 2004) | | Parent |
|  | Discipline style: praise and incentives (Parenting Practices Interview, PPI) | (Webster-Stratton, 1998b; Webster-Stratton et al., 2004) | | Parent |
|  | Discipline style: clear expectations (Parenting Practices Interview, PPI) | (Webster-Stratton, 1998b; Webster-Stratton et al., 2004) | | Parent |
| Child behavior | Problem behavior (Problem scale, Eyberg Child Behavior Inventory, ECBI) | (Eyberg & Ross, 1978) | | Parent |
|  | Problem behavior: intensity (Intensity scale, Eyberg Child Behavior Inventory, ECBI) | (Eyberg & Ross, 1978) | | Parent |
|  | Problem behavior (Total difficulties scale, Strengths and Difficulties Questionnaire, SDQ) | (Goodman, 1997; van Widenfelt, Goedhart, Treffers, & Goodman, 2003) | | Parent |
|  | Problem behavior: conduct problems (Conduct problems scale, Strengths and Difficulties Questionnaire, SDQ) | (Goodman, 1997; van Widenfelt et al., 2003) | | Parent |
|  | Problem behavior: hyperactivity and inattention (Hyperactivity and inattention scale, Strengths and Difficulties Questionnaire, SDQ) | (Goodman, 1997; van Widenfelt et al., 2003) | | Parent |
|  | Problem behavior: aggressive behavior (adapted version, Teacher Rating of Aggression) | (Dodge & Coie, 1987; Hendrickx, Crombez, Roeyers, & Orobio de Castro, 2003) | | Parent |
| Parental perspectives | Parenting stress (Parenting Stress Index) | (Abidin, 1983; De Brock, Vermulst, Gerris, & Abidin, 1992) | | Parent |

| **Matsumoto et al. (2007)** | | |  | |
| --- | --- | --- | --- | --- |
| **Outcome name** | **Assessment instrument** | **Information about instrument** | | **Reported by** |
| Parenting behavior | Parenting practices: laxness (laxness scale, Parenting Scale, PS) | (Arnold et al., 1993) | | Parent |
|  | Parenting practices: overreactivity (overreactivity scale, Parenting Scale, PS) | (Arnold et al., 1993) | | Parent |
|  | Parenting practices: verbosity (verbosity scale, Parenting Scale, PS) | (Arnold et al., 1993) | | Parent |
| Child behavior | Problem behavior (problem scale, Eyberg Child Behavior Inventory, ECBI) | (Eyberg & Pincus, 1999) | | Parent |
|  | Problem behavior: intensity (intensity scale, Eyberg Child Behavior Inventory, ECBI) | (Eyberg & Pincus, 1999) | |  |
| Parental perspectives | Parental confidence (Problem Settings and Behavior Checklist, PSBC) | (Sanders & Woolley, 2005) | | Parent |

| **Taylor et al. (1997)** | | | |
| --- | --- | --- | --- |
| **Outcome name** | **Assessment instrument** | **Information about instrument** | **Reported by** |
| Parenting behavior | Parent-child interactions (sensitivity to cues scale, response to infant distress scale, social-emotional growth fostering scale, clarity of cues scale, responsiveness of the child to the parent scale, The Nursing Child Assessment Teaching Scale, NCATS) | (Barnard, Hammond, Booth, Bee, & Spieker, 1989) | Observer |
|  | Environment to stimulate cognitive development (emotional and verbal responsibility scale, avoidance of restriction and punishment scale, provision of appropriate play materials scale, opportunities for variety of daily stimulation scale, HOME) | (Bradley & Caldwell, 1976; van Doorninck, Caldwell, Wright, & Frankenburg, 1981) | Observer |
| Child behavior | Mental development (mental development index, Bayley Scales of Infant Development) | No reference cited in article | Observer |
|  | Psychomotor development (psychomotor development index, Bayley Scales of Infant Development) | No reference cited in article | Observer |
| Parental perspectives |  |  |  |

| **Turner et al. (2007)** | | |  | |
| --- | --- | --- | --- | --- |
| **Outcome name** | **Assessment instrument** | **Information about validation** | | **Reported by** |
| Parenting behavior | Parenting practices: laxness (laxness scale, Parenting Scale, PS) | (Arnold et al., 1993) | | Parent |
|  | Parenting practices: overreactivity (overreactivity scale, Parenting Scale, PS) | (Arnold et al., 1993) | | Parent |
|  | Parenting practices: verbosity (verbosity, Parenting Scale, PS) | (Arnold et al., 1993) | | Parent |
| Child behavior | Problem behavior (problem scale, Eyberg Child Behavior Inventory, ECBI) | (Eyberg & Pincus, 1999) | | Parent |
|  | Problem behavior: intensity (intensity scale, Eyberg Child Behavior Inventory, ECBI) | (Eyberg & Pincus, 1999) | | Parent |
|  | Problem behavior (total difficulties, Strengths and Difficulties Questionnaire - extended version, SDQ) | (Goodman, 1999; Moos & Moos, 1986) | | Parent |
|  | Problem behavior: total impact (total impact scale, Strengths and Difficulties Questionnaire - extended version, SDQ) | (Goodman, 1999; Moos & Moos, 1986) | | Parent |
| Parental perspectives |  |  | |  |

| **Webster-Stratton (1998)** | | |  | |
| --- | --- | --- | --- | --- |
| **Outcome name** | **Assessment instrument** | **Information about instrument** | | **Reported by** |
| Parenting behavior | Discipline style: harsh (Oregon Social Learning Centers Discipline Questionnaire, OSLC) | Adaptation of OSLC Discipline Questionnaire, revised for preschoolers to include discipline style and techniques (no reference cited in article) | | Parent |
|  | Discipline style: consistent (Oregon Social Learning Centers Discipline Questionnaire, OSLC) | No reference cited in article | | Parent |
|  | Discipline style: positive (Oregon Social Learning Centers Discipline Questionnaire, OSLC) | No reference cited in article | | Parent |
|  | Discipline style: appropriate limit setting (Daily Discipline Interview, DDI) | (Webster-Stratton & Spitzer, 1991) | | Observer |
|  | Discipline style: physical negative discipline techniques (Daily Discipline Interview, DDI) | (Webster-Stratton & Spitzer, 1991) | | Observer |
|  | Discipline style: verbal negative discipline techniques (Daily Discipline Interview, DDI) | (Webster-Stratton & Spitzer, 1991) | | Observer |
|  | Parent-child interactions: positive parenting (Dyadic Parent-Child Interactive Coding System Revised, DPICS-R) | (Webster-Stratton & Spitzer, 1991) | | Observer |
|  | Parent-child interactions: total critical statements (Dyadic Parent-Child Interactive Coding System Revised, DPICS-R) | (Webster-Stratton, 1985a) | | Observer |
|  | Parent-child interactions: total commands (Dyadic Parent-Child Interactive Coding System Revised, DPICS-R) | (Webster-Stratton, 1985a) | | Observer |
|  | Parent-child interactions: valence (Dyadic Parent-Child Interactive Coding System Revised, DPICS-R) | (Webster-Stratton, 1985a) | | Observer |
|  | Parent-child interactions: nurturing or supportive (Coder Impression Inventory, CII) | Adaptation of OSLC Impression Inventory (no reference cited in article) | | Observer |
|  | Parent-child interactions: harsh or critical (Coder Impression Inventory, CII) | No reference cited in article | | Observer |
|  | Parent-child interactions: discipline condemnation (Coder Impression Inventory, CII) | No reference cited in article | | Observer |
| Child behavior | Social skills: positive social behavior (Social Competence Scale) | Conduct Problem Prevention Research Group (no reference cited in article) | | Parent |
|  | Problem behavior (Child Behavior Checklist, CBCL) | (Achenbach & Edelbrock, 1991) | | Parent |
|  | Problem behavior (total problem scale, Eyberg Child Behavior Inventory, ECBI) | (Robinson et al., 1980) | | Parent |
|  | Parent-child interactions: deviance and noncompliance (Dyadic Parent-Child Interactive Coding System Revised, DPICS-R) | (Webster-Stratton, 1985a) | | Observer |
|  | Parent-child interactions: total positive affect and prosocial behavior (Dyadic Parent-Child Interactive Coding System Revised, DPICS-R) | (Webster-Stratton, 1985a) | | Observer |
|  | Parent-child interactions: valence (Dyadic Parent-Child Interactive Coding System Revised, DPICS-R) | (Webster-Stratton, 1985a) | | Observer |
|  | Parent-child interactions: misbehavior and negative affect (Coder Impression Inventory, CII) | Adaptation of OSLC Impression Inventory (no reference cited in article) | | Observer |
|  | Parent-child interactions: positive affect and prosocial behavior (Coder Impression Inventory, CII) | No reference cited in article | | Observer |
|  | Parent-child interactions: overall poor conduct (Coder Impression Inventory, CII) | No reference cited in article | | Observer |
| Parental perspectives |  |  | |  |

| **Webster-Stratton (2001)** | | |  | |
| --- | --- | --- | --- | --- |
| **Outcome name** | **Assessment instrument** | **Information about validation** | | **Reported by** |
| Parenting behavior | Negative parenting |  | |  |
|  | Parent-child interactions: harsh (variable harsh style, Parenting Practices Inventory, PPI) | Adaptation of Oregon Social Learning Center’s discipline questionnaire, revised for young children (no reference cited in article) | | Parent |
|  | Parent-child interactions: negative/critical (Coder Impressions Inventory, CII) | Adaptation from the Oregon social Learning Center Impression Inventory (no reference cited in article) | | Observer |
|  | Parent- child interactions: total critical statements (Dyadic Parent-Child Interactive Coding System-Revised, DPICS-R) | (Webster-Stratton, 1989) | | Observer |
|  | Positive parenting |  | |  |
|  | Parenting practices: positive parenting (one variable from the LIFT parenting practices interview) | Adaptation of Oregon Social Learning Center’s discipline questionnaire, revised for young children (no reference cited in article) | | Parent |
|  | Parenting practices: monitoring (one variable from the LIFT parenting practices interview) | No reference cited in article | | Parent |
|  | Parenting practices: frequency of activities with child (one variable from the Parent Involvement Questionnaire, INVOLVE-P) | Scale derived from the Oregon Social Learning Center questionnaire (no reference cited in article) | | Parent |
|  | Parent-child interactions: emotional and cognitive stimulation (Coder Impressions Inventory, CII) | Adaptation from the Oregon social Learning Center Impression Inventory (no reference cited in article) | | Observer |
|  | Parent-child interactions: positive affect, praise and physical warmth (Dyadic Parent-Child Interactive Coding System-Revised, DPICS-R) | (Webster-Stratton, 1989) | | Observer |
| Child behavior | Conduct problems |  | |  |
|  | Problem behavior: intensity (intensity scale, Eyberg Child Behavior Inventory, ECBI) | (Robinson et al., 1980) | | Parent |
|  | Problem behavior (Child Behavior Checklist, CBCL) | (Achenbach & Edelbrock, 1991) | | Parent |
|  | Parent-child interactions: percent time child acts inappropriate (Coder Impressions Inventory, CII) | Adaptation from the Oregon social Learning Center Impression Inventory (no reference cited in article) | | Observer |
|  | Parent-child interactions: deviance, noncompliance, no opportunity (Dyadic Parent-Child Interactive Coding System-Revised, DPICS-R) | (Webster-Stratton, 1989) | | Observer |
| Parental perspectives |  |  | |  |

Table ii Details of the coding for the moderator adaptations

| **Study** | **Cultural sensitivity** | **Process of adaptation** |
| --- | --- | --- |
| Bjørknes et al. 2013 | Deep (and surface) structure sensitivity   - Sessions directly translated into Somali/Urdu by bilingual assistants - Somali/Urdu versions of parent material - Mother-only groups - Extensive use of role play in Somali/Urdu versions of parent material - Specialized content: expanded focus on large sibling groups and emotion control. | Detailed information   - The study reports what adaptations were made to the parent training program, and referred to a pilot study for more information |
| Brotman et al. 2011 | Deep (and surface) structure sensitivity   - A well-known male African American television personality is the narrator of video segments - Specialized content: two sessions that focus on cultural and contextual influences on parenting and child development - Culture was explicitly discussed in each group | No to little information   - The study reports a combination of activities regarding adaptations of the program (e.g. informed by prevention science, developmental literature, input and collaboration from community stakeholders, parents and teachers), but no specific details were given on how this resulted in the adaptations made |
| Coard et al. 2007 | Deep (and surface) structure sensitivity   - Use of African American language expression, common language - Emphasis on African American values about collective responsibility, cooperation, and interdependence - Use of African proverbs, sayings and affirmations, poems, quotes, symbols and pledges - Use of prayer, role-playing, storytelling, extended family participation, and humor - Use of a setting and motif representative of the population - Group leaders are African American - Specialized content: racial socialization | Detailed information   - Study refers to recent literature on parenting challenges of African American families and details were given on the specific content added to an empirically supported standard program |
| Day et al. 2012 | Surface structure sensitivity   - Local parents as peer facilitators | No to little information |
| Fagan et al. 2002 | Deep (and surface) structure sensitivity   - All male staff, training of fathers to become facilitators - Specialized content: racism in society, negative images of African American men, racial socialization, and the effect of racism on fathering | No to little information   - The study reports a combination of activities regarding adaptations of the program (e.g. literature, empowerment practice approach), but no specific details were given on how this resulted in the adaptations made |
| Ghosh Ippen 1998 | Surface structure sensitivity   - Program was led in Spanish and English - Program materials translated in Spanish | No to little information |
| Gottfredson et al. 2006 | Basic sensitivity | No to little information |
| Gross et al. 2003 | Surface structure sensitivity   - At least one group leader was from the same ethnic background as the majority of parents in the group | No to little information |
| Gross et al. 2009 | Surface structure sensitivity   - At least one group leader was from the same ethnic background as the majority of parents in the group - Videotaped scenes in parents' homes and public spaces to portray situations that are familiar relevant and stressful for parents; multi-ethnic display of ethnicities in videos | Detailed information   - Information on the development of the parent training program in collaboration with a parent advisory group. The authors reported what advise was given by the advisory group and how this was incorporated in the program to make it culturally and contextually relevant for ethnic minority and low-income parents. |
| Kim et al. 2008 | Surface structure sensitivity   - Program materials translated to Korean - Program delivered in Korean | No to little information |
| Kim et al. 2014 | Deep (and surface) structure sensitivity   - English and Korean program materials - Group leaders are bilingual and bicultural - Specialized content: cultural elements and parenting practices that parents perceive to be effective (e.g. a common physical punishment method used by Korean parents, biblical principles, Korean parenting virtues) | Detailed information   - Multiple theories were used to bring together cultural elements and parenting practices that were relevant for the target population, and examples are given on how this resulted in adaptations |
| Lau et al. 2011 | Surface structure sensitivity   - Program delivered in Mandarin and Cantonese - Specialized content was encompassed in five sessions: cognitive restructuring, communication training, positive proactive parental involvement | No to little information |
| Leijten et al. 2015 | Surface structure sensitivity   - Interpreters were present during intervention sessions - Mother-only groups - Pictures added to homework assignments to make it easier to understand for mothers with limited language skills | No to little information |
| Matsumoto et al. 2007 | Surface structure sensitivity   - Translated materials - Japanese group leaders | No to little information |
| Taylor et al. 199 | Basic sensitivity | No to little information |
| Turner et al. 2007 | Surface structure sensitivity   - Each group was co-facilitated by one indigenous health worker | No to little information   - Study reports that adaptations were made with extensive community consultation, but no specific details were given on how this resulted in the adaptations made |
| Webster-Stratton 1998 | Surface structure sensitivity   - Four parents were trained as group leaders - Families shown on videotapes came from a variety of ethnic and socioeconomic background | No to little information |
| Webster-Stratton et al. 2001 | Surface structure sensitivity   - Program translated in Vietnamese and Spanish - Program delivered by trained leaders in Vietnamese and Spanish - Families shown on videotapes came from a variety of ethnic and socioeconomic backgrounds | No to little information |

**References**

Abidin, R. R. (1983). *Parenting stress index manual*. Charlottesville, Virgina: Pediatric Psychology Press.

Abidin, R. R. (1995). *Parenting Stress Index Professional Manual 3rd edition*: Psychological Assessment Resources.

Achenbach, T. M., & Edelbrock, C. S. (1991). *Manuel for the Child Behavior Checklist and Revised Child Behavior Profile*. Burlington, Vermont: University Associates in Psychiatry.

Achenbach, T. M., & Rescorla, L. A. (2001). *Manual for the ASEBA school-age forms and profiles*. Burlington: University of Vermont.

Arnold, D. S., O'Leary, S. G., Wolff, L. S., & Acker, M. M. (1993). The parenting scale: a measure of dysfunctional parenting in discipline situations. *Psychological Assessment, 5*(2), 137-144.

Barnard, K. E., Hammond, M. A., Booth, C. L., Bee, H. L., & Spieker, S. J. (1989). Measurement and meaning of parent-child interaction. *Journal of Applied Developmental Psychology, 3*, 39-80.

Bradley, R. H., & Caldwell, B. M. (1976). The relationship of infants' home environments to mental test performance at fifty-four months: a follow-up study. *Child Development, 47*, 1172-1174.

Brotman, L. M., Gouley, K. K., Chesir-Teran, D., Dennis, T., Klein, R. G., & Shrout, P. (2005). Prevention for preschoolers at high risk for conduct problems: immediate outcomes on parenting practices and child social competence. *Journal of Clinical Child & Adolescent Psychology, 34*(4), 724-734.

Brotman, L. M., Gouley, K. K., Huang, K. Y., Kamboukos, D., Fratto, C., & Pine, D. S. (2007). Effects of a psychosocial family-based preventive intervention on cortisol response to a social challenge in preschoolers at high risk for antisocial behavior. *Archives of General Psychiatry, 64*(10), 1172-1179.

Calzada, E. J., & Brotman, L. M. (2002). Effective Parenting Test (EPT). *Unpublished assessment instrument*.

Chamberlain, P., & Reid, J. B. (1987). Parent observation and report of child symptoms. *Behavioral Assessment, 9*, 97-109.

Choe, H. S., & Chung, O. B. (2010). A study on validity of the Parenting Efficacy Scale: A base study on development of the scale. *Korean Journal of Child Studies, 22*, 1-16.

De Brock, A. A. J. L., Vermulst, A. A., Gerris, J. R. M., & Abidin, R. R. (1992). *Nijmeegse ouderlijke stress index, handleiding experimentele versie [Nijmegen parenting stress index, manual experimental version]*. Amsterdam, the Netherlands: Swets & Zeitlinger.

Dodge, K. A., & Coie, J. D. (1987). Social-information-processing factors in reactive and proactive aggression in children's peer groups. *Journal of Personality and Social Psychology, 53*(6), 1146-1158.

Eyberg, S., & Pincus, D. (1999). *Eyberg Child Behavior Inventory and Sutter-Eyberg Student Behavior Inventory-Revised. Professional manuel*. Florida: PAR.

Eyberg, S., & Robinson, E. (2000). *Dyadic parent-child interaction coding system: Manuel*. Seattle, Washington: University of Washington.

Eyberg, S., & Ross, A. W. (1978). Assessment of child behaviour problems: the validation of a new inventory. *Journal of Clinical Child Psychology, 7*(2), 113-116.

Goodman, R. (1997). The Strengths and Difficulties Questionnaire: a research note. *Journal of Child Psychology and Psychiatry, 38*(5), 581-586.

Goodman, R. (1999). The extended version of the Strengths and Difficulties Questionnaire as a guide to child psychiatric caseness and consequent burden. *Journal of Child Psychology & Psychiatry, 40*(5), 791-799.

Gottfredson, D. C., Harmon, M., Gottfredson, G. D., Jones, E. M., & Celestin, J. A. (1996). *Compendium of prevention program outcomes and instrument locator*. Ellicott City, Maryland: Gottfredson Associates, Inc.

Gottman, J., Declaire, J., & Goleman, D. (1998). *Raising an emotionally intelligent child*. New York: Simon & Schuster.

Gresham, F. M., & Elliot, S. N. (1990). *Social Skills Rating System*. Circle Pines, Minnesota: American Guidance Service.

Gross, D., Conrad, D., Fogg, L., Willis, L., & Garvey, C. (1995). A longitudinal model of maternal self-efficacy, depression, and difficult temperament during toddlerhood. *Research in Nursing & Health, 44*, 96-101.

Gross, D., & Rocissano, L. (1988). Maternal confidence in toddlerhood: Its measurement for clinical practice and research. *Nurse Practitioner, 13*, 19-29.

Hendrickx, M., Crombez, G., Roeyers, H., & Orobio de Castro, B. (2003). Psychometrische evaluatie van de Nederlandstalige versie van de Agressie Beoordelingsschaal van Dodge en Coie [Psychometric evaluation of the Dutch version of the Agression Scale by Dodge and Coie]. *Gedragstherapie, 36*.

Jellinek, M. S., & Murphy, J. M. (1990). The recognition of psychosocial disorders in pediatric office practice: the current status of the pediatric symptom checklist. *Journal of Developmental & Behavioral Pediatrics, 11*(5), 273-278.

Kellam, S. G. (1990). Developmental epidemiological framework for family research on depression and aggression. In G. R. Patterson (Ed.), *Depression and aggression in family interaction*. Hillsdale, New Jersey: Lawrence Erlbaum.

Kim, E., Guo, Y., Koh, C., & Cain, K. (2010). Korean immigrant discipline and children's social competence and behavior problems. *Journal of Pediatric Nursing, 25*, 490-499.

MacPhee, D., Benson, J. B., & Bullock, D. (1986). *Influences on maternal self-perceptions*. Paper presented at the Fifth Biennial International Conference on Infant Studies, Los Angeles, California.

McCabe, K. M., Clark, R., & Barnett, D. (1999). Family protective factors among urban African American youth. *Journal of Clinical Child Psychology, 28* (2), 137-150.

Moos, R. D., & Moos, B. S. (1986). *Family environment scales manuel*. Palo Alto, California: Consulting Psychologists Press Inc.

Prinz, R. J., Foster, S., Kent, R. N., & O'Leary, K. D. (1979). Multivariate assessment of conflict in distressed and nondistressed mother-adolescent dyads. *Journal of Applied Behaviora Analysis, 12*(4), 691-700.

Reynolds, C. R., & Kamphaus, R. W. (1992). *Manual for the Behavioral Assessment System for Children (BASC).* Circle Pines, Minnesota: American Guidance Service.

Reynolds, C. R., & Kamphaus, R. W. (2002). *The clinician's guide to the Behavior Assessment System for Children (BASC)*. New York: Guilford.

Robinson, E. A., Eyberg, S., & Ross, A. W. (1980). The standardization of an inventory of child conduct problem behaviors. *Journal of Clinical Child Psychology, 9*, 22-29.

Rohner, R. (1991). *Handbook for the study of parental acceptance and rejection*. Connecticut: University of Connecticut: Storrs.

Sanders, M. R., & Woolley, M. L. (2005). The relationship between maternal self-efficacy and parenting practices: implications for parent training. *Child: Care, Health and Development, 31*(1), 65-73.

Scott, S., Spender, Q., Doolan, M., Jacobs, B., & Aspland, H. (2001). Multicentre controlled trial of parenting groups for childhood antisocial behaviour in clinical practice. *BMJ, 323*(7306), 194-198.

Shelton, K. K., Frick, P. J., & Wootten, J. (1996). Assessment of parenting practices in families of elementary schoolage children. *Journal of Clinical Child Psychology, 24*, 317-329.

Stevenson, H. C. (1994). Validation of the scale of racial socialization for African American adolescents: Steps towards multidimensionality. *Journal of Black Psychology, 20*, 445-468.

Stevenson, H. C. (1997). Rationale for the measurement of racial socialization beliefs and experiences. *Unpublished manuscript, University of Pennsylvania, Philadelphia*.

van Doorninck, W. J., Caldwell, B. M., Wright, C., & Frankenburg, W. K. (1981). The relationship between twelve-month home stimulation and school achievement. *Child Development, 52*(3), 1080-1083.

van Widenfelt, B. M., Goedhart, A. W., Treffers, P. D. A., & Goodman, R. (2003). Dutch version of the Strengths and Difficulties Questionnaire (SDQ). *European Child & Adolescent Psychiatry, 12*(6), 281-289.

Webster-Stratton, C. (1985a). Comparison of abusive and nonabusive families with conduct-disordered children. *American Journal of Orthopsychiatry, 55*(1), 59-69.

Webster-Stratton, C. (1985b). Mother perceptions and mother–child interactions: Comparison of a clinic-referred and non-clinic group. *Journal of Clinical Child Psychology, 14*, 334-339.

Webster-Stratton, C. (1989). Dyadic Parent-Child Interaction Coding System - Revised. *Unpublished manuscript, University of Washington, Seattle*.

Webster-Stratton, C. (1998a, 1998). Parent Practices Interview. Retrieved from http://www.ssb.no/english/subjects/02/01/10/innvbef_en/.

Webster-Stratton, C. (1998b). Preventing conduct problems in Head Start children: strengthening parenting competencies. *J Consult Clin Psychol, 66*(5), 715-730.

Webster-Stratton, C. (2007a). Parent practice interview. Retrieved from http://www.son.washington.edu/centers/parenting-clinic/.

Webster-Stratton, C. (2007b). Social Competence Scale. Retrieved from http://www.son.washington.edu/centers/parenting-clinic/.

Webster-Stratton, C., Reid, M. J., & Hammond, M. (2004). Treating children with early-onset conduct problems: intervention outcomes for parent, child, and teacher training. *Journal of Clinical Child & Adolescent Psychology, 33*(1), 105-124.

Webster-Stratton, C., & Spitzer, A. (1991). Development, reliability and validity of the Daily Telephone Discipline Interview: DDI. *Behavioral Assessment, 13*, 221-239.

1. References are cited such as reported in the studies [↑](#footnote-ref-1)
